# Effect of *Origanum vulgare* Hydroalcoholic Extract on *Giardia lamblia* Cysts Compared with Metronidazole in Vitro

**Published:** 2018

**Authors:** Jaber DAVOODI, Saeid ABBASI-MALEKI

**Affiliations:** 1. Dept. of Microbiology, Faculty of Medical and Sciences, Zanjan Branch, Islamic Azad University, Zanjan, Iran; 2. Dept. of Pharmacology and Toxicology, Urmia Branch, Islamic Azad University, Urmia, Iran

**Keywords:** Anti-Giardia activity, Cyst, Hydroalcoholic extract, Metronidazole

## Abstract

**Background:**

Giardiasis, an intestinal infection, is made by the flagellate protozoan and on the other hand, positive effects of plants derivatives, especially phenolic derivatives, against giardiasis. The effect of *Origanum vulgare* (OV) hydroalcoholic extract is still uninvestigated. Thus, this study was conducted to evaluate the effect of OV hydroalcoholic extract on *Giardia lamblia* cysts compared with metronidazole in vitro.

**Methods:**

The present experimental study was conducted in 2015–2016 in the Laboratory of Department of Parasitology of Islamic Azad University (Abhar Branch, Abhar, Iran). Cysts separated from feces by Bingham procedure were calculated by using the Hemusytumetr method. Five hundred μl of concentrations of 10, 100 and 200 mg/ml of OV hydroalcoholic extract and also125 mg/kg of metronidazole were added to the purified cysts of giardia. Control group was treated with normal saline. Anti-Giardia activity was calculated by using the light microscope for 30, 60 and 120 min and after exposure to eosin stain.

**Results:**

The results indicated anti-Giardia activity of OV hydroalcoholic extract and the best response was achieved at higher levels so that there were no significant differences among OV groups at levels of 200 mg/kg with metronidazole (*P*>0.05).

**Conclusion:**

The anti-Giardia activity of Origanum *vulgare* extract is may due to the presence of phenolic compounds present in it.

## Introduction

Giardiasis, one of the most common parasitic infections, has extensively found in humankind and *Giardia lamblia* is usually caused it (1–3). The increasing spread of giardiasis is caused major concerns all over the world and especially in developing countries, i.e. Iran. In giardiasis, cysts are a major factor for transmission of this parasitic infection. The cysts usually tolerate severe pH and temperature and the feces of infected host is the main source for their transmission. Giardiasis usually transmits in human through ingestion of cysts in water and feed (4). Giardiasis, after several months, can create malabsorption and weight loss which subsequently disturb immune system and finally death (5–7). The different drugs have been used for the treatment of giardiasis, such as tinidazole, albendazole, and furazolidone, but metronidazole has extensively used (8–10). Previous studies have been documented undesirable effects of these drugs (8, 9). Considering the Metronidazole as common drug for treatment of giardiasis, it may cause loss of appetite, vomiting, diarrhea, lethargy, weakness, anemia, blood in the urine, head tilt, seizures, disorientation, stumbling (4), liver diseases, nervous disorders, and its mutagenic effect in some bacteria and animal models (8, 11,12).

In the past years, medicinal plants and their derivatives have interested more attention, as a safe alternative, because they have the extensive range of metabolites with potential therapeutic value (13, 14). Broad ranges of the population in developing countries are depended on medicinal plants to supply the health (15, 16).

*Origanum vulgare* (OV: oregano) is belonging to the *Lamiaceae* family and it has been extensively used, because of antioxidant properties (17, 18). Oregano, for several years, has been used as folk medicine (19). Methanolic extract of oregano, because of phenolic compounds present in the extract, scavenges free radicals (20). Antibacterial activity of oregano has been extensively shown for an extensive range of bacteria (21, 22). Oregano, because of strong antioxidant and antibacterial properties, may show anti-Giardia activity and also be replaced with metronidazole.

Thus, the present study was conducted to evaluate the effect of *Origanum vulgare* hydroalcoholic extract on G. *lamblia* cysts compared with metronidazole in vitro.

## Materials and Methods

The present experimental, laboratory study was conducted in 2015–2016 in the Laboratory of Department of Parasitology of Islamic Azad University (Abhar Branch, Abhar, Iran).

### Preparation of Origanum vulgare

Fresh and flowering aerial parts of OV were collected from Sahand Mountain slopes (Maragheh, Iran) in spring 2015 and immediately transferred to herbarium for identification of compounds by the botanist in Maragheh University. The prepared samples were dried by using an oven at a temperature of 41°C and then fined to moderate powders (290–650 μm) by the mill.

### Preparation of hydroalcoholic extract

The prepared powders of OV (75 g) were extracted using maceration and percolation by 75% ethanol for three times consecutively in both extractive processes. The prepared solution was concentrated by using a rotary evaporator which lowered pressure. The residues water was re-suspended in pH 7.1 phosphate buffered solution (PBS). These solutions were sterilized using filtration by a 0.24-μm membrane and kept at sterile bottles at 5 °C until analysis. The phenolic compounds of the present in OV extract were identified (23) (Table 1). The phenolic flavonoid compounds present in the samples were identified on the basis of their UV and mass spectra and retention times in comparison with commercial standards and the results are presented in mg/g of extract. Moreover, the samples were analyzed for several times and data presented as mean.

**Table 1:** Phenolic and flavonoid compounds present in OV extract

***Compounds***	***Amount (mg/g extract)***
3-*O*-Caffeoylquinic acid	0.4
Protocatechuic acid	0.68
5-*O*-Caffeoylquinic acid	1.09
Apigenin 6,8-di-*C*-glucoside	0.73
Kaempferol *O*-hexosyl-*O*-hexoside	0.27
Myricetin 3-*O*-glucoside	0.65
Quercetin *O*-hexoside	0.49
4-[[(2′,5′ Dihydroxybenzoyl)oxy]methyl]phenyl O-β-D-glucopyranoside	4.71
Taxifolin	0.85
Quercetin 3-*O*-rutinoside	5.21
Quercetin 7-*O*-hexoside	0.81
Luteolin *O*-glucuronide	14.05
Luteolin 7-*O*-glucoside	23.15
Apigenin 7-*O*-rutinoside	2.75
Rosmarinic acid	17.51
Apigenin 7-*O*-glucuronide	6.43
Kaempferol *O*-hexoside	1.14
Kaempferide *O*-glucuronide	2.75
Eridictyol	1.15
Lithospermic acid A	2.75
Methylapigenin *O-*glucuronide	1.47
Naringenin	0.98

### Separation of G. lamblia cysts

The *G. lamblia* cysts obtained from freshly excreted feces of patients referred to parasitology laboratory and they were immediately processed.

The cysts were separated (24). Five grams of feces samples were mixed with 10 ml physiological serum for 30 min and subsequently filtered by 4-layer filter. The samples were subsequently centrifuged for 3 min at 1500 rpm. The upper solution was removed and then 10 ml sucrose solution (2M) was added to the residue and centrifuged for 10 min at 1800 rpm. The upper solution containing cyst was separated and 10 ml normal saline (0.9%) was added to above solution and centrifuged for 5 min at 1000 rpm. Finally, 2 ml from the lower part tube was kept until future analysis on the basis recommendations (25).

### Investigation of effects of hydroalcoholic extract on cysts of G. lamblia

The different concentrations of hydroalcoholic extract, 10, 100 and 200 mg/ml, were prepared by using DMSO (dimethyl-sulfoxide) solvent. Five hundred μl of different concentration of extract and 125 mg/kg of metronidazole (Alborz-Daru Pharmaceutical Co, Tehran-Iran), were separately mixed with suspensions containing cyst for different times, 30, 60 and 120 min and were subsequently exposed to 500 μl of 1% of eosin stain (25). Part of samples was served as control and treated with saline. The light microscope was used for the count of cysts and the number of the cysts was expressed as the percentage. The active and dead cysts were observed in red color and colorless, respectively (24).

### Statistical analysis

The results expressed as the mean ± standard error of the mean (S.E.M). All data were analyzed statistically using one-way analysis of variance (ANOVA), followed by a Tukey-test. Differences with *P*<0.05 were considered statistically significant. All statistical analyses were also performed using Prism with version 7 (GraphPad Software, Inc., San Diego, CA, USA).

## Results

**Fig. 1:**
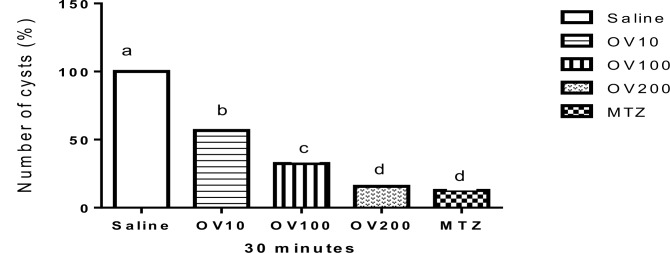
Comparison of the anti-Giardia activity of different concentration of *Origanum vulgare* (OV) hydroalcoholic extract and metronidazole (MTZ) for 30 min. Superscripts (a–d) show significant differences (*P*<0.001) among treatments

**Fig. 2:**
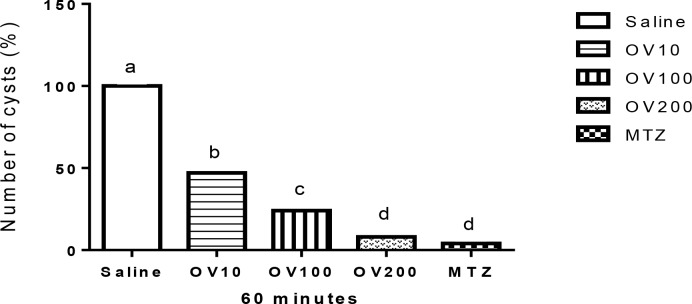
Comparison of the anti-Giardia activity of different concentration of *Origanum vulgare* (OV) hydroalcoholic extract and metronidazole (MTZ) for 60 min. Superscripts (a–d) show significant differences (*P*<0.001) among treatments.

**Fig. 3:**
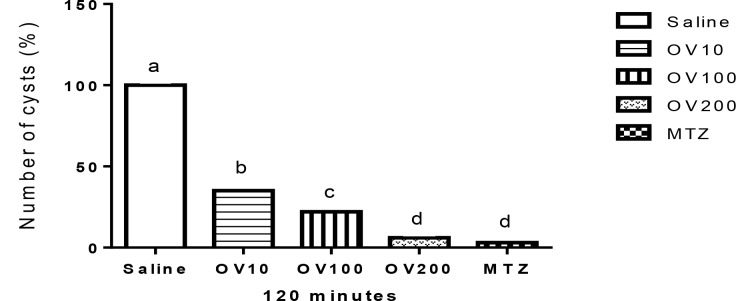
Comparison of the anti-Giardia activity of different concentration of *Origanum vulgare* (OV) hydroalcoholic extract and metronidazole (MTZ) for 120 min. Superscripts (a–d) show significant differences (*P*<0.001) among treatments

Figures 1–3 show the effect of OV extract and metronidazole on Giardia cysts for 30, 60 and 120 min, respectively. The best response for anti-Giardia activity was found at higher levels of extract, so that there were not there were no significant differences among OV group at levels of 200 mg/kg with Metronidazole (*P*>0.05) at all times. In addition, the level of 100 mg/ml of the extract showed better activity compared with the level of 10 mg/ml.

**Fig. 4:**
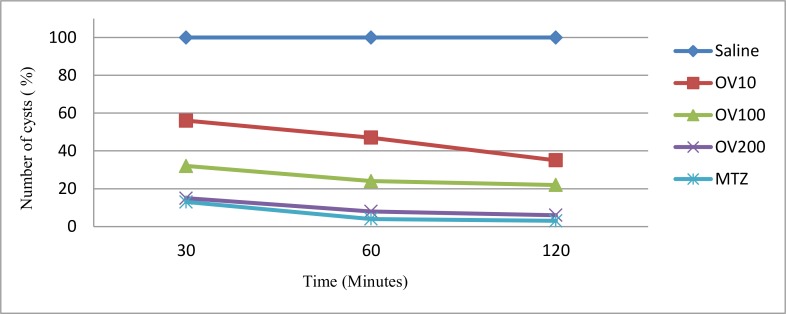
Comparison of the effect of different concentration of *Origanum vulgare* (OV) hydroalcoholic extract and metronidazole (MTZ) at different times

Figure 4 shows the effect of OV extract and Metronidazole for different times. The extract at the levels of 100 and 200 mg/ml and Metronidazole approximately showed stable activity during 30–120 min.

The lowest level of OV extract showed better activity at longer times; showing that longer times would be needed to improve the anti-Giardia activity at lower levels.

## Discussion

The *G. lamblia*, an intestinal parasite, is especially known by diarrhea in humans and especially in infants (9, 26). Different drugs (such as tinidazole, albendazole, furazolidone, and metronidazole), have been used for the treatment of giardiasis (9, 26). These drugs, especially Metronidazole, can cause loss of appetite, vomiting, diarrhea, lethargy, weakness, and anemia, blood in the urine, head tilt, seizures, disorientation and stumbling (4).

Researchers are interested in using medicinal plants for the treatment of different diseases since they usually did not create any disorder at optimum levels. In the current study, OV extract, especially at high levels could show anti-giardiasis activity. The lower levels also showed anti-*Giardia* activity, so that lowest level showed better activity at longer times; suggesting that lowest level would be required to more times for activity. It has been shown beneficial effects of some plant derivates against *G. lamblia* cysts and trophozoites under in vitro and in vivo conditions (16, 27, 28). Plant extracts prevented proliferation, and adherence *G. lamblia* to the intestine and digestive system (16, 29, 30). We could not find any study investigating the effect of OV extract/essential oil on giardiasis, but the anti-bacterial activity of OV at levels of similar to our study, 100 and 200 mg/ml has been reported (21–22). Phenolic compounds present in essential oils and extracts show antimicrobial effect.

Considering the relation between medicinal plants and parasites, some compounds present in plant extracts, especially phenolic compounds, not only may inhibit membrane development and respiration, but also elevate fluidity and permeability in parasites (31). The use of essential oils firstly increased Giardia peripheral vesicles and subsequently removed it (32). Our results (Table 1) showed that phenolic compounds are present in OV extract and it seems to be responsible for anti-Giardia activity and also it can be stated that higher levels, because of high phenolic compounds, can vastly interact with cysts membrane.

## Conclusion

Our results well showed the anti-Giardia activity of OV extract, especially at high levels, however, lowest level efficiently showed its activity at longer times. It can be suggested to use OV hydroalcoholic extract for anti-Giardia activity and also as an alternative for metronidazole.
